# Anthralin Suppresses the Proliferation of Influenza Virus by Inhibiting the Cap-Binding and Endonuclease Activity of Viral RNA Polymerase

**DOI:** 10.3389/fmicb.2020.00178

**Published:** 2020-02-18

**Authors:** Ao Hu, Jing Li, Wei Tang, Ge Liu, Haiwei Zhang, Chunlan Liu, Xulin Chen

**Affiliations:** ^1^State Key Laboratory of Virology, Wuhan Institute of Virology, Chinese Academy of Sciences, Wuhan, China; ^2^University of Chinese Academy of Sciences, Beijing, China; ^3^Guangdong Key Laboratory of Virology, Institute of Medical Microbiology, Jinan University, Guangzhou, China

**Keywords:** influenza, cap snatching, vRdRp, anthralin, cap-binding, endonuclease

## Abstract

Influenza virus RNA-dependent RNA polymerase (vRdRp) does not have capping activity and relies on the capped RNAs produced by the host RNA polymerase II (RNAPII). The viral polymerases process the capped RNAs to produce short capped RNA fragments that are used as primers to initiate the transcription of viral mRNAs. This process, known as cap-snatching, can be targeted by antiviral therapeutics. Here, anthralin was identified as an inhibitor against influenza a virus (IAV) infection by targeting the cap-snatching activity of the viral polymerase. Anthralin, an FDA-approved drug used in the treatment of psoriasis, shows antiviral activity against IAV infection *in vitro* and *in vivo*. Importantly, anthralin significantly reduces weight loss, lung injury, and mortality caused by IAV infection in mice. The mechanism of action study revealed that anthralin inhibits the cap-binding function of PB2 subunit and endonuclease activity of PA. As a result, viral mRNA transcription is blocked, leading to the decreases in viral RNA replication and viral protein expression. In conclusion, anthralin has been demonstrated to have the potential of an alternative antiviral against influenza virus infection. Also, targeting the captive pocket structure that includes the N-terminus of PA endonuclease domain and the C-terminal of PB2 cap-binding domain of IAV RdRp may be an excellent strategy for developing anti-influenza drugs.

## Introduction

Influenza A viruses (IAV) are enveloped viruses of the family *Orthomyxoviridae* that cause contagious respiratory diseases and substantial morbidity and mortality (Palese, 2007). Since the IAV genome is composed of segmented RNAs, which makes it more susceptible to antigenic drift and shift, more strains have undergone resistance mutations against traditional antiviral treatment (Palese, 2007; Krammer et al., 2018). As the risk of influenza pandemic continues to increase, it becomes increasingly important to understand the interactions between drug candidates and targets. We urgently need to develop new antiviral strategies to improve therapeutic effectiveness, especially in the case of severe diseases, and are less vulnerable to antiviral resistance (Loregian et al., 2014).

Currently, three classes of antiviral drugs were approved by the FDA for the treatment and prophylaxis of influenza (De Clercq, 2006). The M2 inhibitors, amantadine and rimantadine blocks the activity of M2 proteins that are ion channels, thereby preventing the viral genomic fragments from entering the cytoplasm (Pinto and Lamb, 2007). The prevalence of viruses resistant to these drugs has been growing around the world, greatly undermining effectiveness of drugs. All currently circulating IAV strains are resistant to M2 inhibitors, so these drugs are no longer recommended for flu treatment (Shiraishi et al., 2003; Bright et al., 2006; Garten et al., 2009). The second class of influenza drugs is neuraminidase inhibitors (NAI), including oseltamivir, peramivir, and zanamivir. NA inhibitors preventing the release of the progeny virus particles through binding to the NA and obstructing its enzymatic activity. Since oseltamivir does not inhibit influenza virus replication, it is not effective in inhibiting the inflammation caused by the virus replication, leading to a poor prognosis. A rapid rise in oseltamivir resistance was detected amongst seasonal A/H1N1 isolates before the 2009 pandemic (Renaud et al., 2011). The third class of anti-influenza drugs is RNA polymerase inhibitors. Favipiravir (T-705), a new drug currently in the clinical trial, acts as a purine analog, inhibits multiple RNA viruses by targeting viral polymerase. But it increases uric acid, causes digestive system diseases and has reproductive toxicity (Furuta et al., 2005; Sangawa et al., 2013; Furuta et al., 2017). Compared with NAI, viral RNA polymerase inhibitors have higher tolerance and longer treatment time, so they may be more effective. The most recently approved drug for influenza virus polymerase is baloxavir marboxil (trade name Xofluza). It is a medication that has been developed by Shionogi, a Japanese pharmaceutical company, for the treatment of influenza A and influenza B. The drug prevents the proliferation of influenza virus by inhibiting the endonuclease activity of the PA subunit (Omoto et al., 2018). However, according to the clinical results, compared with treatment in adults, baloxavir showed weak therapeutic effect in the susceptible population that includes children, adolescents and the elderly, which cannot shorten the course of the disease (Hayden et al., 2018; Kakuya et al., 2019). The specific amino acid substitution at the PA active endonuclease site (I38T/F) could decrease the sensitivity of the variant virus strain to baloxavir by 11 to 57 times. In the third phase of the trial, 9.7% of the baloxavir-treated patients were found with the variant virus with I38T/M/F replacement in PA decreased the susceptibility to baloxavir, sometimes related to the rebound of virus titer and the continuous prolongation of symptoms (Hayden et al., 2018). Therefore, new efforts need to be made to discover and develop new flu drugs with improved properties. Adverse reactions, such as hematochezia, after taking baloxavir have been reported (Kanai et al., 2019). Nevertheless, the synthesis of viral mRNA, cRNA, and vRNA is the central event and crucial for influenza virus life cycle. Both host factors and virus polymerase are involved in this process, during which, cap-binding, endonuclease, and vRdRp activity, are attractive targets for small molecule inhibitors. In fact, the viral polymerase of influenza virus is the target for several novel anti-influenza candidates under active clinical development (McKimm-Breschkin et al., 2018).

Anthralin (also known as dithranol), is a synthetic version of a natural compound found in goa powder from the araroba tree. This medication is used for the treatment of long-term psoriasis by decreasing the number of DNA-synthesizing cells in the psoriatic epidermis and the keratinization process, but this decrease is not explicit in other cells and conditions (Liden and Lesson, 1974). Previously, anthralin was identified in our lab to inhibit the replication of a broad spectrum of influenza viruses in both MDCK and A549 cells (An et al., 2014). In 2015, Roch et al. (2015) reported that Endo-1, an analog of anthralin, has a potent antiviral effect by inhibiting the endonuclease activity of influenza virus polymerase. In this study, we tested the antiviral activity in mice model and found that anthralin reduces the weight loss, lung injury, and mortality caused by lethal IAV infection. The mechanism of action study suggests that anthralin acts on the RNA replication stage of the virus life cycle, which includes the transcription of viral mRNAs and the replication of viral RNAs. To further define the targets of anthralin, we investigated the functions of viral and host factors associated with virus RNA transcription and replication. We found that anthralin prevents the PB2 from binding to the capped RNAs and the cleavage of capped RNAs mediated by the endonuclease activity of PA. Taken together, we demonstrated that anthralin has the potential to serve as a new type of influenza virus RdRp inhibitor with inhibitory effects on both cap-binding and endonuclease activities.

## Materials and Methods

### Virus and Cells

Influenza virus strain A/Puerto Rico/8/1934 (H1N1) virus (PR8) was provided by the virus collection center at Wuhan Institute of Virology, Chinese Academy of Sciences, China. The Madin–Darby Canine Kidney (MDCK) cells (ATCC CCL-34) and HEK293T cells were cultured in Dulbecco’s modified Eagle’s medium (DMEM; Gibco, Invitrogen). The Human Pulmonary Epithelial (A549) cells (ATCC CCL-185) were maintained in Roswell Park Memorial Institute medium 1640 (RPMI1640; Gibco, Invitrogen). Both DMEM and RPMI1640 were supplemented with 10% fetal bovine serum (FBS; Gibco, Invitrogen), and 100 U/ml penicillin, 100 U/ml streptomycin. All these cells were maintained at 37°C in a 5% CO_2_ incubator.

### Chemicals

Anthralin and 5,6-dichlorobenzimidazole (DRB) were purchased from Aladdin (Aladdin Industrial Corporation, Shanghai, China) and the 50 mM drug stock solution was formulated with the dimethyl sulfoxide. Ribavirin was purchased from Sigma Chemical Company (Sigma-Aldrich, St. Louis, MO, United States) and a 100 mM stock solution was made for use. ApG was purchased from Jena Bioscience.

### Plasmids

The pT7CEF1-NHis-GST-CHA expression vector provided by the 1-step CHO High-Yield IVT Trial Kit acellular expression system (Thermo Fisher Scientific). Open reading frames encoding PA, PB1, and PB2 were amplified by PCR. Recombinant proteins of vRdRp were expressed with pT7CEF1-NHis- GST-CHA.

### Antibodies

Rabbit anti-PA, anti-PB1, anti-PB2, and anti-NP antibodies were purchased from GeneTex Inc. (GeneTex, United States). Mouse monoclonal antibodies against influenza A virus HA and M2 were obtained from Santa Cruz Biotechnology (Santa Cruz, CA). Anti-glyceraldehyde-3-phosphate dehydrogenase (GAPDH) was purchased from Beyotime Biotechnology. The antibodies of human RNA pol II, RNA pol II CTD phosphor Ser2 and RNA pol II CTD phosphor Ser5 were purchased from Active Motif company.

### Cytotoxicity and Antiviral Assays

Madin–Darby Canine Kidney or A549 cells were seeded in 96-well plates and cultured for 24 h at 37°C. Then the cells were treated with anthralin serially diluted with fresh medium and incubated at 37°C for 72 h. Compound toxicity was determined by AlamarBlue^®^ Assay (Invitrogen). Three independent experiments were performed in duplicate for the calculation of 50% cell cytotoxic concentration (CC_50_) using Prism v.6 software.

For antiviral assay, in the presence of eight concentrations of 2-fold serial dilutions from 200 μM, MDCK cells were plated and infected with PR8 at an MOI of 0.01, or A549 cells were plated and infected with PR8 at an MOI of 1. After incubation at 37°C for 48 h, the inhibition of viral replication was measured by the modified neuraminidase activity (NA) assay (Ivachtchenko et al., 2013). The fluorescence intensity was measured with a multi-label plate reader (Wallac Envision, PerkinElmer, MA, United States) and was expressed as the 50% effective (inhibitory) concentrations (EC_50_). For detection of infectious virus yield reduction, the supernatants harvested 48 hpi were titrated through the TCID_50_ assay, and the virus titers were calculated according to the method of Spearman–Karber. Ribavirin was used as a positive control.

### Time-of-Addition Assay

The time-of-addition assay was conducted using the method modified from that originally described (An et al., 2014). In the pretreatment assays, the PR8 viruses were treated with 50 μM of anthralin at room temperature for 1 h, before infection with PR8 virus at MOI of 0.01 at 4°C for 1 h followed by incubation at 37°C for 10 h. Then, the cell supernatants were harvested for detection of the production of infectious virus particles by reinfection. For determining the stage of the virus life cycle that is inhibited, the cells were precooled at 4°C for 30 min, followed by infection with PR8 virus at 4°C for 1 h. Then cells were incubated at 37°C by adding anthralin at different times. The cell supernatants were harvested 10 hpi for detection of the production of infectious virus particles by reinfection.

### Indirect Immunofluorescence Assay

MDCK and A549 cells were planted in 96-well plates and cultured for 24 h at 37°C. Cells were inoculated with medium alone (negative control), PR8, or virus incubated with various concentrations of the anthralin at 4°C for 1 h. Then the cells were washed three times with cold PBS and inoculated with medium for 24 h at 37°C. Cells were fixed with 4% paraformaldehyde for 20 min at room temperature. The cells were then incubated with IFA buffer (PBS containing 3% BSA, 0.3% Triton X-100 and 10% FBS) for 1 h and incubated with a primary antibody against the NP for 1 h. The cells were then washed with PBS and incubated with FITC-conjugated rabbit anti-mouse secondary antibody (1:1000). The stained samples were then examined with a Nikon’s Eclipse Ti inverted microscope (Nikon, Tokyo, Japan).

### Western Blotting

A549 cells were planted into a six-well plate and incubated overnight. Cells were precooled at 4°C for 30 min and then infected with influenza virus PR8 at MOI of 5 for 1 h at 4°C for synchronizing viral infections. After washing with cold PBS, the cells treated with or without 50 μM anthralin, and then harvested at the indicated times after infection. Total cells were lysed in radio-immunoprecipitation assay (RIPA) buffer and separated on 10% sodium dodecyl sulfate-polyacrylamide gel electrophoresis (SDS-PAGE), and then samples were transferred to polyvinylidene difluoride membranes. After membranes were incubated with primary antibody at 1:2000 dilution at 4°C for overnight, the appropriate secondary antibody at 1:10000 dilution was added and incubated. The proteins were detected using an enhanced chemiluminescence Western blotting detection system and antibodies directed against proteins of IAV, cell, and GAPDH.

### Fluorescence Microscopy and Image Analysis

In time-course infection experiments, cells grown on glass-bottom dishes (Cellvis formerly *In vitro* Scientific, Mountain View, CA, United States) were chilled on ice and washed with cold PBS. A549 cells were infected with PR8 virus at an MOI of 1 and synchronized at 4°C for 1 h. The cells were then washed three times with cold PBS, and the cells were fixed with 4% paraformaldehyde in PBS at 48 h post-transfection. Cells were permeabilized with 0.1% Triton X-100 in PBS, blocked in 5% bovine serum albumin (Sigma) in PBS, and incubated with antibodies against influenza virus proteins. After washing with PBS, the cells were incubated with Alexa Fluor488 goat anti-rabbit, Alexa Fluor594 goat anti-mouse and DAPI for 1 h.

To detect co-localization of the vRdRp and RNAPII, A549 cells were infected with PR8 virus at an MOI of 10 at 4°C for 1 h, washed three times with PBS, and then treated with or without 50 μM anthralin. The cells were harvested 9 h after infection, fixed with 4% paraformaldehyde for 20 min, and permeabilized with 0.5% Triton in PBS for 3 min at room temperature. Cells were incubated with rabbit anti-PA, anti-PB1, anti-PB2 primary antibody overnight and then incubated with the Alexa Fluor 594-conjugated goat anti rabbit IgG and Alexa Fluor 488-conjugated goat anti-mouse IgG for 1 h. After washing, confocal images were acquired with a 60 × objective, using a Nikon A1 confocal microscope with random sampling.

### Quantitative RT-PCR of Virus RNAs

Total RNA and mRNA were extracted from cells using TRIzol reagent (Invitrogen) at the indicated time and the first strand of cDNA was synthesized using SuperScript III Reverse Transcriptase (Invitrogen) and reverse-transcribed with the following primers: 5′-GAGAGAGGAGAAGAGACA-3′ for PA vRNA, 5′-AGTAGAAACAAGGTACTTTTTTGGAC-3′ for PA cRNA, 5′-GACGATGCAACGGCTGGTCTG-3′ for NP vRNA, 5′-AGTAGAAACAAGGGTATTTTTCTTTA-3′ for NP cRNA, 5′-GTCGAAACGTACGTTCTCTCTATC-3′ for M2 vRNA, 5′-AGTAGAAACAAGGTAGTTTTTTACTC-3′ for M2 cRNA, oligo(dT)20 for viral mRNA, and 5′-GGTGTGTTACAAAGGGC AGGG-3′ for 18S rRNA. Single-stranded cDNAs were then subjected to quantitative real-time PCR by using SYBR green (Roche, Germany) with following specific primer sets: 5′-GAGA GAGGAGAAGAGACA-3′ and 5′-TTAATTTTAAGGCATCCA TCAGCAGG-3′ for PA, 5′-AGCATTGTTCCAACTCCTTT-3′ and 5′-GACGATGCAACGGCTGGTCTG-3′ for NP, 5′-GTCG AAACGTACGTTCTCTCTATC-3′ and 5′-TCCCCTTAGTCAG AGGTGAC-3′ for M2, and 5′-AACGGCTACCACATCCAAGG-3′ and 5′-GGGAGTGGGTAATTTGCGC-3′ for 18S rRNA.

### Luciferase Reporter Assay

HEK293T cells were transfected with luciferase reporter plasmid pGL6-vRdRp-lucwhich containing vRdRp response elements and Renilla control plasmid pRL-SV40-N using Lipofectamine 3000 transfection reagent (Invitrogen, Carlsbad, CA, United States). At 48 h after co-transfection, HEK293T cells were lysed and Luciferase activity was analyzed by the Dual-Luciferase Reporter Gene Assay Kit (Beyotime Biotechnology) according to the manufacturer’s protocol.

### Expression and Purification of Influenza Virus Polymerase

Influenza virus polymerase proteins were expressed by using the 1-step CHO High-Yield IVT Trial Kit acellular expression system (Thermo Fisher Scientific) according to the manufacturer’s protocols. The plasmids expressing full length PA, PB1, and PB2 were constructed, respectively, using pT7CEF1-NHis-GST-CHA expression vector provided by the kit. The expressed proteins were purified using GST purification kit (Beyotime Biotechnology). The expression and purification of the polymerases were analyzed by SDS-PAGE and Western blot.

### Gel Shift Assay

For the vRNA binding assays, 0.5 μM polymerase complexes were incubated in binding buffer with 0.5 μM 5′ FAM-labeled vRNA (5′-FAM-AGUAGAAACAAGGCC-3′) and different concentrations of anthralin at 37°C for 30-min. For cap-binding assay, following a 30-min reaction for the binding of 0.5 μM polymerase complexes with an unlabeled oligoribonucleotide containing the chemically synthesized 0.5 μM vRNA, the 0.5 μM 20-nucleotide capped RNA (5′-FAM-m^7^GpppAAUCUAUAAUAGCAUUAUCC-3′) was added, and the mixture was incubated in the presence of anthralin at 37°C for 30 min. These protein-RNA complexes were separated from free RNA by electrophoresis on 4% non-denaturing gels.

### Endonuclease Assay

This experiment was conducted as described (Pflug et al., 2014). We used fluorescence labeling instead of isotope labeling. We have separated synthesized vRNA, which was an equimolar mixture of nucleotides 1–15 from the 5′ end (5′-AGUAGAAACAAGGCC-3′) and nucleotides 1–18 from the 3′ end (3′-UCGUCUUCGUCUCCAUAU-5′). 0.5 μM vRdRp or PA, 0.5 μM vRNA and 0.5 μM capped RNA were incubated in the reaction buffer (150 mM NaCl, 50 mM HEPES, pH 7.5, 5 mM MgCl_2_ and 2 mM TCEP) at 37°C, and 30 min later, the dilution series of anthralin were added to the experimental group and incubated at 37°C for 1 h. The samples were analyzed on a 20% acrylamide, 7 M urea denaturing gel.

### Animal Experiment

All experiments were conducted according to the protocol approved by the Animal Care and Use Committee of Wuhan Institute of Virology of the Chinese Academy of Sciences (WIVA08201801). BALB/c mice (6–8 weeks) were purchased from the Beijing Vital River Laboratory Animal Technology Co., Ltd. (Charles River laboratories China). Mice were anesthetized with pentobarbital sodium and then infected with the influenza virus through the nasal cavity. Infected mice were treated by intragastric managed 50 and 25 mg/kg/d of anthralin once a day for five consecutive days starting 4 h after infection. On the third day after the infection, the lungs of mice were inflation fixed in 10% formalin and paraffin-embedded and then stained with H&E. At the same time, mouse tracheas and lungs were removed and washed twice with PBS containing 0.1% BSA, then centrifuged at 10000 × *g* for 10 min at 4°C. Supernatants of the bronchoalveolar lavage fluids (BALFs) were then harvested for viral titer determination using the endpoint dilution assay in MDCK cells as described above.

### Statistical Analysis

Data are represented as mean ± SD. For all analyses, multiple independent experiments (*N* ≥ 3) were carried out. The differences in survival (as compared to control) were tested using two-way ANOVA tests. Log-rank test were used for survival. Student’s *t*-tests were performed for the level of significance. GraphPad Prism 6 (GraphPAD Software Inc.) software was used for data analysis and preparation of all graphs. Statistical significance was determined by two-tailed *P-*values: ns *P* > 0.05, ^∗^*P* < 0.05, ^∗∗^*P* < 0.01, ^∗∗∗^*P* < 0.001.

## Results

### Anthralin Inhibits Influenza Virus Replication *in vitro* and *in vivo*

**FIGURE 1:**
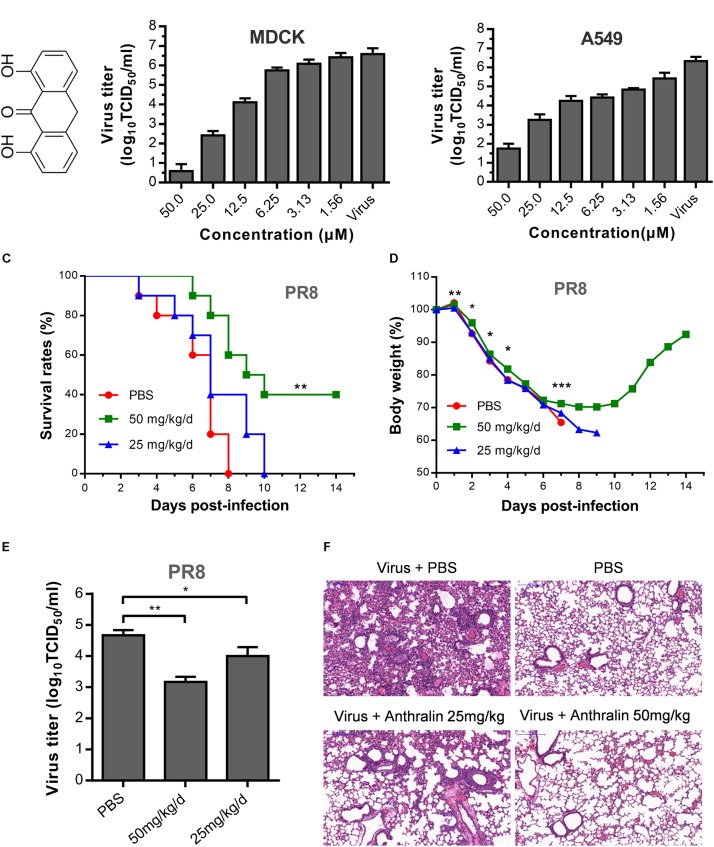
The antiviral activity of anthralin *in vitro* and *in vivo*. **(A)** Chemical structures of anthralin. **(B)** The antiviral effects of anthralin on the production of infectious virions on two cell lines. The supernatants were harvested and the virus titers were determined by TCID_50_ assay. Virus titers were calculated according to Spearman–Karber method as described in methods. The values represent the means ± S.D. of duplicate samples from three independent experiments. **(C)** Effect of anthralin administration on survival rates in the infected mice. BALB/c mice were infected with 1000 TCID_50_ of PR8 and treated with anthralin or PBS control (*n* = 10). **P < 0.01 with respect to PBS controls. **(D)** Weight loss of mice infected with lethal influenza virus and treated with or without anthralin. *P < 0.05, **P < 0.01 and ***P < 0.001 with respect to PBS controls. **(E)** Virus titers in BALFs taken on the third day after infection were measured by TCID_50_ assay determined on MDCK cells. **(F)** Haematoxylin & Eosin-stained sections from lungs of non-infected and PR8-infected BALB/c mice treated with anthralin or PBS at 3 dpi (day post infection) (scale = 200 μm).

Previously, we identified anthralin as an active antiviral agent against influenza virus by screening an FDA approved drug library using MDCK cells. Anthralin (Figure 1A) was found to inhibit all six subtypes of influenza A virus and one influenza B virus tested (An et al., 2014). To further study the mechanism of action of anthralin on influenza viruses, we tested further the effect of anthralin in the production of infectious influenza viruses in MDCK and A549 cells. The results showed that the virus titers were significantly reduced concentration-dependently upon anthralin treatment, resulted in a 99% reduction at 12.5 μM and more than 99.99% reduction at 50 μM in both cell lines (Figure 1B). Anthralin is not toxic in A549 and MDCK cells at up to 50 μM (Supplementary Figure S1).

Next, the *in vivo* therapeutic effect of anthralin was evaluated in the mouse model. In the assessment of the toxicity of anthralin, the maximum dosage was determined to be 50 mg/kg/d (data not shown). BALB/c mice were challenged with a lethal dose (4LD_50_) of PR8 virus, a mouse-adapted IAV strain, and then treated with 25 and 50 mg/kg/d of anthralin in phosphate-buffered saline (PBS) or just PBS. Our results showed that high-dose anthralin treatment reduced the mortality compared to that of PBS control and low-dose treatment (Figure 1C). Forty percent of the mice that gained body weight in the 50 mg/kg/d of anthralin treated group survived (Figure 1D). For determining the virus titers in the lungs of the influenza virus infected mice, the BALFs were harvested on day three post-infection for virus titer determination. The results showed that compared with PBS control, 50 mg/kg/d of anthralin treatment reduced the virus titers by greater than 90% in the BALFs, while 25 mg/kg/d of anthralin treatment reduced the virus titers by 70% in the BALFs (Figure 1E). The pathological changes in the lungs of infected mice treated or not treated with anthralin were examined by hematoxylin and eosin staining. The results showed that the pulmonary parenchyma and interstitial inflammatory infiltrations of the mice with anthralin treatment were improved in both treated groups compared with the PBS group, it was greatly improved in the high dose group (Figure 1F).

Therefore, these results suggested that anthralin can efficiently inhibit the replication of influenza virus *in vivo*, by lowering the virus titers, decreasing the pathological effects mediated by the inflammatory infiltration of the lungs and protect mice against lethal influenza A virus infection. Taken together, we confirmed that anthralin inhibits influenza virus replication *in vitro* and *in vivo*.

### Anthralin Inhibits Influenza Virus Replication on the Mid-Stage of Its Life Cycle

**FIGURE 2:**
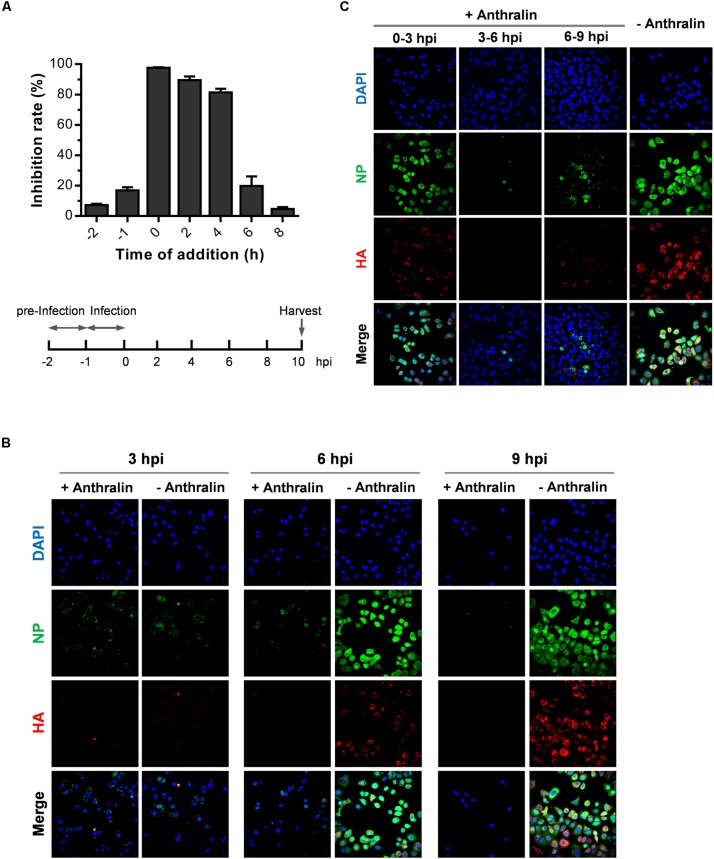
Anthralin inhibits the mid-phase of the influenza life cycle. **(A)** MDCK cells were precooled at 4°C for 30 min. (i) Cells were incubated with anthralin at 4°C for 1 h before infected with PR8 virus at 4°C for 1 h, the NA activities in the supernatants were measured 10 hpi. (ii) Cells were incubated with virus and anthralin at 4°C for 1 h, the NA activities in the supernatants were measured 10 hpi. (iii) Time-of-addition assay: Cells were infected with PR8 virus at an MOI of 0.01 at 4°C for 1 h, anthralin was added at an interval of 2 h after virus adsorption. The NA activities in the supernatants were measured 10 hpi. **(B,C)** A549 cells were precooled at 4°C for 1 h, and infected with PR8 virus at an MOI of 5 at 4°C for 1 h to synchronize the infection. The infected cells were treated with anthralin at 37°C until being fixed at 3, 6, and 9 hpi., respectively, **(B)**, or were treated with anthralin at different time periods and the cells were fixed 9 hpi **(C)**, and stained with anti-NP, HA and 4′,6-diamidino-2-phenylindole (DAPI).

To identify the stage of the influenza virus life cycle that anthralin acts on, we tested the time-dependent inhibitory effects of anthralin on influenza virus replication using a time-of-addition assay as described previously (An et al., 2014). Anthralin was added at different times, and the cells were harvested 10 h post-infection (hpi). MDCK cells pretreated with anthralin at 4°C for 1 h and then infected by IAV (PR8 strain at MOI of 0.01) at 4°C. Then cells were washed twice with PBS and cultured in the absence of anthralin. The influenza virus replication was found not affected by the pretreatment of cells by anthralin. When the PR8 virus was pretreated with anthralin at 4°C for 1 h and then used to infect MDCK cells, it replicates as efficient as the untreated (data not shown). These results suggest that anthralin does not have a virucidal effect on the influenza virus or inhibitory effect on virus adsorption. Whereas, anthralin could efficiently inhibit the virus replication when added during the early to mid-stage of the virus life cycle, indicating that the anthralin affects the entry or the virus replication stage (Figure 2A).

To investigate which stage of influenza replication is inhibited by anthralin treatment, we monitored the expression and localization of virus NP and HA on the influenza virus infected cells that were treated by anthralin for different times or untreated by confocal fluorescence microscopy analysis. As shown in Figure 2B in A549 cells synchronously infected by PR8 virus, the internalization of the virus was not affected until 3 hpi. Anthralin treatment for 6 h resulted in reduced expression of NP and HA in A549. However, the nucleus import of NP is not affected. Treatment after virus infection by anthralin for 9 h eliminated the expression of NP and HA in A549 cells. There were virtually no NP or HA visible in the cytoplasm. These results suggest that anthralin acts on the mid-stage of the influenza virus life cycle when the virus is replicating its genomic RNA (Figure 2B and Supplementary Figure S2A).

To further analyze the time-dependent events in influenza virus life cycle that are affected by anthralin treatment, we checked the virus replication status by measuring the expression and localization of virus NP and HA in the synchronized infection of A549 cells with anthralin treatment during different time periods (Figure 2C and Supplementary Figure S2B). The results showed that the anthralin treatment during 0–3 h after the initial infection does not affect the internalization of influenza viruses. The anthralin treatment during 3–6 h did not affect the nuclear import of NP, while the expression of NP in the nucleus was greatly reduced, and the NP and HA proteins in the cytoplasm were not observed. These results suggest that anthralin does not interfere with the import of RNP into the nucleus, but seems to affect the viral RNA replication or translation of viral proteins. The anthralin treatment during 6–9 hpi suppressed less efficiently the expression of HA and NP proteins in the infected cells, indicating that anthralin does not interfere with the late stage of the life cycle of IAV, which includes the assembly and release of viral particles.

In summary, our results suggest that anthralin inhibits the replication of influenza virus in the mid-stage of the virus cycle.

### Anthralin Inhibits the Transcription of the Influenza Virus

**FIGURE 3:**
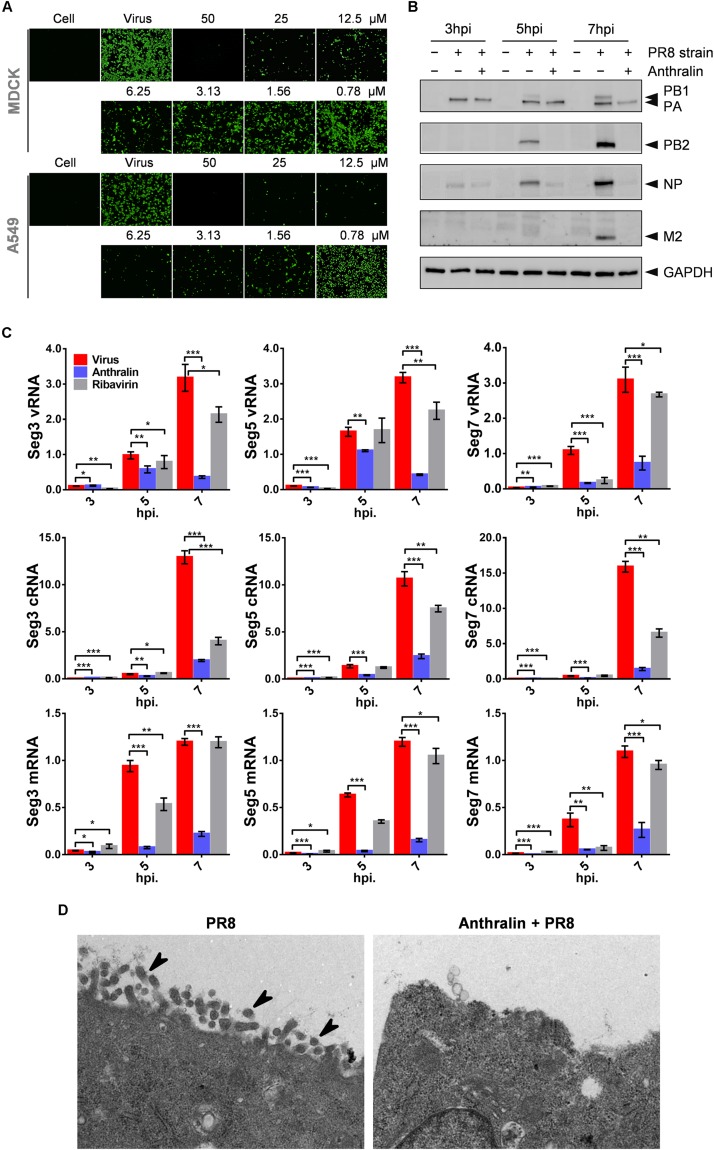
Anthralin inhibits the transcription of the influenza virus and thus interferes with virus replication. **(A)** MDCK cells were infected with PR8 at an MOI of 0.1 and A549 cells were infected with PR8 at an MOI of 1 and treated with serially diluted anthralin starting from 50 μM. After 24 h of incubation at 37°C, the NP expression was measured by indirect immunofluorescence assay using anti-NP. **(B)** A549 cells were infected with PR8 at an MOI of 5 and viral protein levels were determined by western blotting of the whole-cell lysates collected at the indicated time points. **(C)** A549 cells were infected with PR8 at an MOI of 5 and incubated for 3, 5, and 7 h. Total RNAs were prepared from cells, and quantitative RT-PCRs were carried out with primer sets specific for vRNA, cRNA and mRNA of the PA, NP or M2, respectively. The results were normalized to the level of 18S rRNA. The averages and standard deviations determined in three independent experiments are shown. Error bars, mean ± SD of three experiments (*n* = 3). The level of significance was determined by Student’s *t*-test (*P < 0.05; **P < 0.01; ***P < 0.001). **(D)** Influenza virus infected and anthralin-treated or untreated A549 cells were immobilized 8 h after infection. Viral particles inside and outside of the cells were observed using transmission electron microscopy (the arrows in the diagram point to virions).

During virus RNA replication, the segmented negative-stranded genomic RNAs of influenza virus serve as templates and are transcribed into mRNAs in the nucleus, and then transported through the nuclear pore into the cytoplasm for translation. Afterward, cRNAs and vRNAs are synthesized and are assembled with newly translated viral proteins to form viral RNP, which is stable without being degraded (Te Velthuis and Fodor, 2016). Finally, the viruses are assembled and released to complete the whole life cycle of IAVs (Pflug et al., 2014; Te Velthuis and Fodor, 2016). To investigate whether anthralin inhibits the down-stream event of the mid-stage of viral lifecycle, we checked the translation of virus proteins using an indirect immunofluorescence assay (IFA). As can be seen in Figure 3A, the NP levels are decreased upon anthralin treatment concentration-dependently in both MDCK and A549 cells. For evaluation of the global effect of anthralin on the expression of IAV proteins, the A549 cells were infected with PR8 at an MOI of 1 at 4°C followed by incubation with or without anthralin at 37°C, and then cells were harvested at 3, 5, and 7 hpi for detection of viral proteins. As shown in Figure 3B, at 3 hpi, only PA and NP were detected, anthralin treatment did not reduce the virus proteins levels of PA or NP, that were from the infected virions. However, at 5 hpi, the viral proteins of PB1, PB2, PA, and NP were all expressed and their levels were significantly reduced upon anthralin treatment. At 7 hpi, all viral proteins accumulated to high levels in the untreated cells, while there is virtually no protein expressed upon anthralin treatment except residual PA that was from the originally infected virions. The reason that other viral proteins were not detected may contribute to the lower efficiency of antibodies of other viral proteins. However, the host protein GAPDH was not affected all the time, suggesting that the anthralin mediated reduction in viral protein levels is not due to cytotoxic effect. Instead, anthralin reduced the levels of viral protein.

Considering that the synthesis of RNAs is the upstream event of translation, which may contribute to the reduction of viral protein levels, we investigated the effects of anthralin in the synthesis of viral mRNAs, cRNAs, and vRNAs of the gene segments 3, 5, and 7 of IAV in infected cells. As shown in Figure 3C, comparing to ribavirin, which inhibited the replication of the influenza virus and did not affect transcription, anthralin could not only significantly reduce the levels of cRNAs and vRNAs, but also the levels of the mRNAs.

As anthralin can affect both IAV RNA synthesis and protein expression, we speculate that the assembly and release of infectious virions can be blocked as well. To test our hypothesis, we observed the production of virions in the infected A549 cells using electron microscope. Our results showed that anthralin treatment dramatically reduced the formation and release of virions (Figure 3D). Taken together, we assume that anthralin inhibits the activity of viral polymerase and reduces the levels of viral RNA replication and the viral protein expression, resulting in a reduction in the production of mature virus particles.

### Anthralin Does Not Inhibit the Capping Activity of Host RNA Polymerase

**FIGURE 4:**
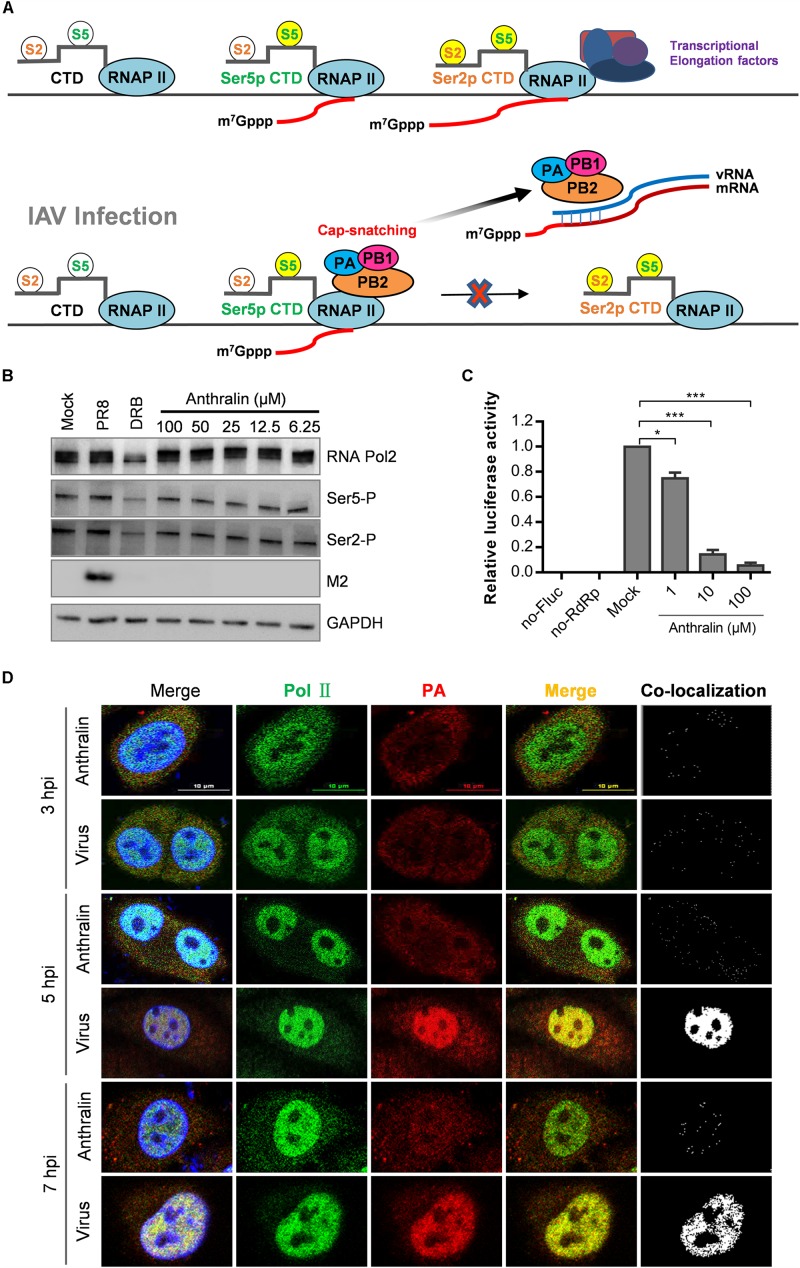
Anthralin does not inhibit the host RNA polymerase activity. **(A)** The model illustrates the phosphorylation of Ser5 and Ser2 of RNAPII (phosphorylated residues are highlighted in bright yellow) and the synthesis of the initial cap structure of transcription. When the cell is infected with the IAV, the viral RdRp complexes complete RNA replication cycle by snatching the caps produced by host RNA II. **(B)** The expression of total RNAPII, serine 2 phosphorylated and serine 5 phosphorylated RNAPII, and the M2 protein of IAV were detected by Western blot. DRB used as a positive inhibitor of transcription initiation (lanes 3). **(C)** Various concentrations of anthralin were incubated with HEK293T cells stably transfected with pGL6-luc (Fluc), pcDNA3.1 expression vector for influenza virus PA, PB1, and PB2 proteins, and pRL-SV40-N (Rluc) as a transfection control, and DMSO was included as the vehicle control. **(D)** A549 cells infected with PR8 virus were treated with 50 μM anthralin during the whole culture process, until being fixed at different time points and the co-localizations of RNAPII and vRdRp were observed using a confocal microscope. The first merge includes PolII, PA and DAPI, whereas the second merge does not include DAPI.

Transcription of the influenza virus is a primer-dependent process that synthesizes the viral mRNAs with a 5′-cap, but the polymerase of IAV does not have inherent capping activity and relies on the capped RNAs produced by the host RNA polymerase II (RNAPII) (Plotch et al., 1981). Therefore, we speculated that anthralin might affect viral transcription by inhibiting the transcriptional competence of RNAPII. The most critical C terminal domain (CTD) residues for RNAPII function are Ser2 and Ser5 (Peterlin and Price, 2006; Harlen et al., 2016). During the transcription of genes, RNAPII complex with high levels of phosphorylation at serine 5 (Ser5P) were enriched during the primal transcription of the gene and capping the nascent RNA, consistent with the important role of Ser5P in the earliest mRNA processing events (Doerks et al., 2002; Schwer and Shuman, 2011). In the subsequent transcriptional extension stage, due to the gradual enrichment and increase of RNAPII containing high levels of phosphor-Ser2 (Ser2P), it is suggested that Ser2P plays an important role in this progress (Barilla‘ et al., 2001; Kim et al., 2004; Fong et al., 2017; Figure 4A).

To test whether anthralin can inhibit the function of host RNAPII, we performed a one-cycle virus replication assay to analyze the phosphorylations of Ser2 and Ser5 of host RNAPII by Western blot. As shown in Figure 4B, as expected, the reference compound DRB (Hensold et al., 1996), a RNAPII CTD kinase inhibitor (Medlin et al., 2003) that can inhibit the transcriptional elongation, reduced the levels of Ser2P and Ser5P significantly. Whereas, the phosphorylation of Ser2 and Ser5 on CTD of RNAPII was not affected by anthralin treatment at all, suggesting that anthralin does not interfere with the activity of host RNAPII. To further understand the transcription and translational processes that are affected by anthralin, we transfected HEK293T cells with the plasmids expressing the pPOLI-Fluc reporter gene under the control of the RNP complex of PR8. The ratios of Fluc to Rluc activity were reduced concentration-dependently upon anthralin treatment, demonstrating that anthralin decreased Fluc activity through inhibiting the vRdRp mediated transcription of pPOLI-Fluc (Figure 4C).

In the process of cap-snatching, the viral polymerase PB2 captures the 5′-cap of nascent host capped RNAs, and the PA cleaves the capped RNA 10–14 nucleotides downstream of the cap structure (Koppstein et al., 2015). The processed cap-containing RNA fragments are then used as primers to initiate transcription of viral mRNAs. Next, the co-localization of influenza virus polymerase complexes and RNAPII in cells that infected with IAV and treated with anthralin for 3, 5, and 7 h were analyzed by fluorescence microscopy. As shown in Figure 4D, at 3 hpi, the host RNAPII localized predominantly in the nucleus, while virus polymerase localized mostly in the cytoplasm. There is a very limited co-localization that may be non-specific random co-localization. During 5–7 hpi, the virus gene transcription and RNA replication are highly efficient and close to complete, host RNAPII and virus polymerase co-localize very well in the nucleus. However, the co-localization of host RNAPII and virus polymerase is completely blocked upon anthralin treatment. Taken together, these results suggest that anthralin inhibits virus RNA dependent RNA polymerase (RdRp) activity not as a result of interference with the function of host RNA polymerase, instead, it may block directly the function of viral RdRp.

### Anthralin Inhibits the Cap-Snatching Activity of Viral RdRp

**FIGURE 5:**
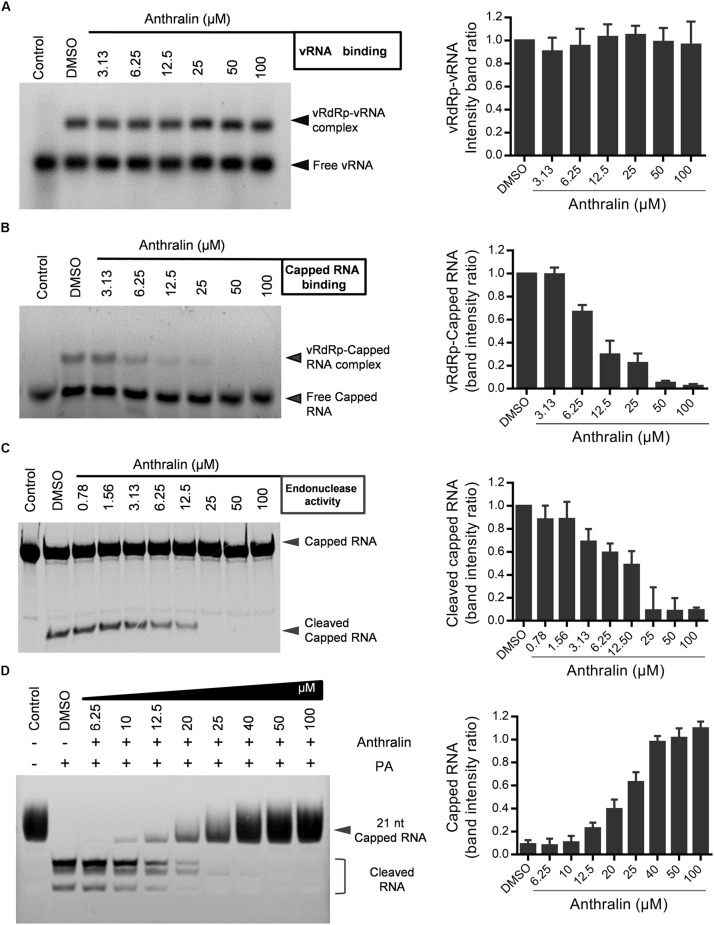
Anthralin inhibits the cap-snatching activity of viral RdRp. **(A)** Binding of vRNA to vRdRp. The polymerase complexes were incubated at 37°C for 30 min in the binding buffer with 5′ FAM-labeled vRNA. The protein-RNA complexes were resolved from free RNA by electrophoresis on 4% non-denaturing gels. **(B)** Cap-binding analysis in a gel shift assay. Following 30 min reaction for the binding of an unlabeled oligoribonucleotide containing the vRNA, the 20-nucleotide-long capped RNAs marked by 5′-FAM were added, and then incubated in the presence or absence of anthralin for 30 min at 37°C. The complexes were separated from free RNA by electrophoresis on 4% non-denaturing gels. **(C,D)** Endoribonuclease assays. 5′ FAM-labeled Capped RNA, vRNA, diluted series anthralin and vRdRp **(C)** or PA subunit alone **(D)** were mixed and incubated with 0.1 μg of tRNA as an additional inhibitor of non-specific nucleases at 37°C water bath for 60 min and samples were analyzed on a 20% acrylamide, 7 M urea denaturing gel.

Since virus transcription relies on the binding of vRNA and capped RNA with vRdRp (Fodor et al., 1994; Tiley et al., 1994; Cianci et al., 1995; Li et al., 1998), we next examined the effects of anthralin on the binding of vRdRp with vRNA and capped RNA, respectively. We found that anthralin inhibited specifically the binding of capped RNA, but not vRNA with viral RdRp (Figures 5A,B). Next, we detected the effects of anthralin on the virus endonuclease activity. We found that anthralin inhibited efficiently and concentration-dependently the cleavage of capped RNA (Figure 5C). Consistent with this, the expressed PA subunit alone has endonuclease function *in vitro* and anthralin can inhibit the endonuclease activity of PA subunit (Figure 5D). In summary, anthralin inhibits the transcription of influenza virus vRNA by inhibiting the cap-binding and endonuclease activities of the viral polymerase and does not directly interfere with the transcription elongation and replication activity of vRdRp. These findings support that the vRdRp is a promising target for antiviral drugs against influenza, which is especially true for the multifunctional vRdRp inhibitors.

## Discussion

Anthralin is a natural anthraquinone derivative, an FDA-approved drug with anti-psoriatic and anti-inflammatory effects. It is used topically in the management of psoriasis, dermatoses, and alopecia areata. Anthralin is also used in biomedical research due to its effect on EGFR autophosphorylation. It controls skin growth by reducing the synthesis of DNA and the mitotic activity in the hyperplastic epidermis, normalizing the rate of cell proliferation and keratinization. There is no current evidence of any long-term anthralin toxicity related either to skin exposure or to systemic issues. In this study, we demonstrated that anthralin inhibits the replication of influenza virus in a mouse model on lowering the virus titers, lung injury, and death rate in mice with lethal IAV infections.

To study the mechanism of action of anthralin against the influenza virus, we performed many assays, including adsorption, entry, nuclear import, and export of RNP, virion production, to assess multiple events in the virus life cycle on which anthralin may affect. We found that anthralin inhibits virus replication in the middle stage of the IAV life cycle, which involves the transcription and replication of the influenza virus genome. Next, we made great efforts to understand the antiviral mechanism of anthralin on RNA replication functions of IAV. Interestingly, in addition to identifying the target of anthralin, our results clarified the timing of transcription and replication of IAV RNA. We found that the reference drug ribavirin inhibits the replication of cRNA and vRNA but not the transcription of viral RNA that have been detected 7 hpi (Figure 3C). While the synthesis of viral mRNA, cRNA, and vRNA are all inhibited by anthralin treatment (Figure 3C), in connection with Figures 2C, 3B, we believe that the anthralin suppresses the cRNA and vRNA synthesis due to the inhibition of transcription of the IAV genome. So, we focused the mechanism study on the effect of anthralin on the transcription functions of viral RdRp.

The influenza virus RdRp cannot synthesize the capped RNA required for transcription initiation. It captures the cap synthesized by the host RNA polymerase II (RNAPII) for initiating transcription of the virus genome (Plotch et al., 1981). In eukaryotes, RNAPII is used for transcription of mRNAs, snRNAs, snoRNAs, and some micro RNAs (Steinmetz et al., 2006). The phosphorylation state of the CTD of RNAPII consisting of tandemly repeated heptapeptides of consensus Tyr1–Ser2–Pro3–Thr4–Ser5–Pro6–Ser7 is required for the spatial and temporal recruitment of various factors are involved in the transcriptional cycle (Jeronimo et al., 2013; Zaborowska et al., 2016). Among the residues in the heptapeptides, the phosphorylation of CTD at Ser2 and Ser5 is involved in the whole process of cap structure synthesis. Different phosphorylation states mark the stage of transcription so that specific cell protein sets can be recruited as needed. The Ser5P CTD modification which is most commonly close to the transcriptional initiation site and still exists with a low abundance can be recognized by cellular mechanisms needed in the preliminary stage of RANPII transcription, such as mRNA capping enzyme (McCracken et al., 1997; Harlen et al., 2016). Influenza virus RdRp can interact directly with RANPII by binding to the CTD with the Ser5P modification. Our results show that anthralin does not inhibit the CTD phosphorylation at Ser2 and Ser5, therefore is unlikely to block the transcriptional initiation of host RNAPII. Then, we studied the effects of anthralin on the functions of viral RdRp.

The influenza virus RdRp is composed of the subunits PB1, PB2, and PA (Te Velthuis and Fodor, 2016). PB1, the N terminus of PB2 (PB2-N), and the C-terminal domain of PA (PA-C) form the core of the polymerase (Te Velthuis, 2019). They are responsible for interacting with the terminal ends of viral promoters and for catalyzing the nucleotidyl transfer reaction. The core also binds the Ser5-phosphorylated CTD (Lukarska et al., 2017; Martin et al., 2018). Influenza RdRp binds to m^7^G on nascent host RNAs through a cap-binding domain on the vRdRp C terminus of PB2 (PB2-C) subunit (Fechter et al., 2003). Then the host RNA at 10–14 nt downstream of the 5′ cap is cleaved by the endonuclease domain at PA-N of vRdRp and form 1- to 3- base pair interactions with the 3′ terminus of the viral genome segment, depending on the sequence of the primer (Dias et al., 2009). This biological process is called “cap-snatching.” The vRdRp displays cap-snatch activity only when bound by vRNA promoter that is formed by the 5′ and 3′ terminal sequences of the vRNA segment (Fodor et al., 1994). Therefore, in order to exclude the effect of vRNA binding on subsequent cap-snatch, which includes cap-binding and cleavage of capped RNA, a vRNA binding assay and a cap-binding assay were performed. Anthralin showed specific inhibition on cap-binding, but not vRNA binding. In addition, anthralin was proved to inhibit the endonuclease activity of vRdRp (Figures 5A–D).

The use of small molecules to affect multiple functions of viral polymerase is beneficial to the development of new antiviral drugs. Our results suggest that anthralin has the potential to be a new anti-influenza drug through a chemical modification to improve its efficacy and to lower its toxicity, and provide a new strategy for the development of novel anti-influenza drugs by targeting dual function of influenza virus polymerase that associated with multiple functions in cap-snatch during viral RNA transcription.

## Data Availability Statement

All datasets generated for this study are included in the article/Supplementary Material.

## Ethics Statement

The animal study was reviewed and approved by the Animal Care and Use Committee of Wuhan Institute of Virology of the Chinese Academy of Sciences.

## Author Contributions

XC and AH conceived and designed the experiments and contributed to the writing of the manuscript. AH, JL, WT, and GL performed the experiments. AH, HZ, CL, and XC analyzed the data.

## Conflict of Interest

The authors declare that the research was conducted in the absence of any commercial or financial relationships that could be construed as a potential conflict of interest.
