# Supplementary figures and images for: Anthralin Suppresses the Proliferation of Influenza Virus by Inhibiting the Cap-Binding and Endonuclease Activity of Viral RNA Polymerase
Source: Front Microbiol. 2020 Feb 18;11:178. doi: 10.3389/fmicb.2020.00178 (PMC7040080; doi:10.3389/fmicb.2020.00178)

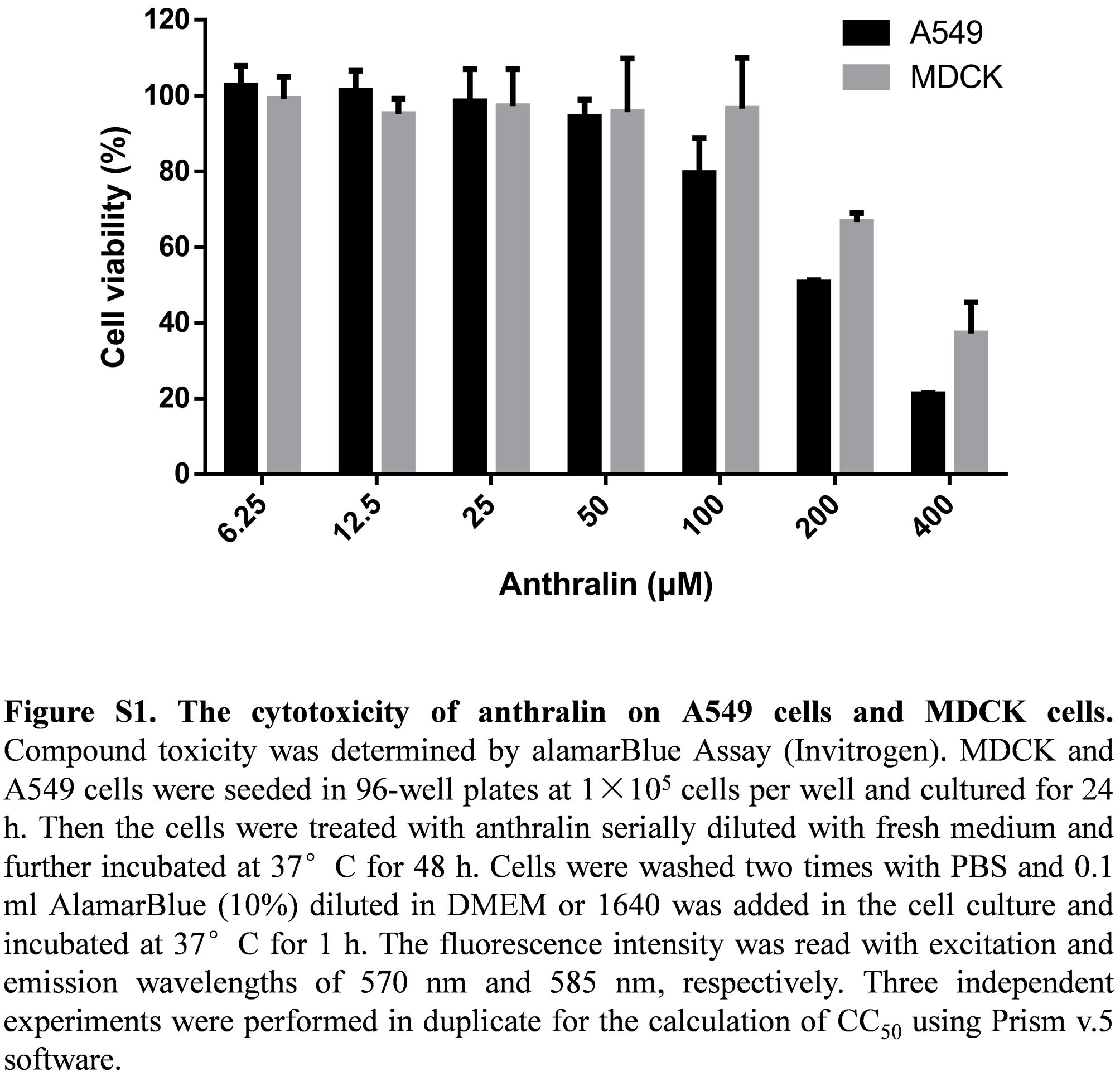

Supplement: Supplementary file 1 [file Image_1.TIF]

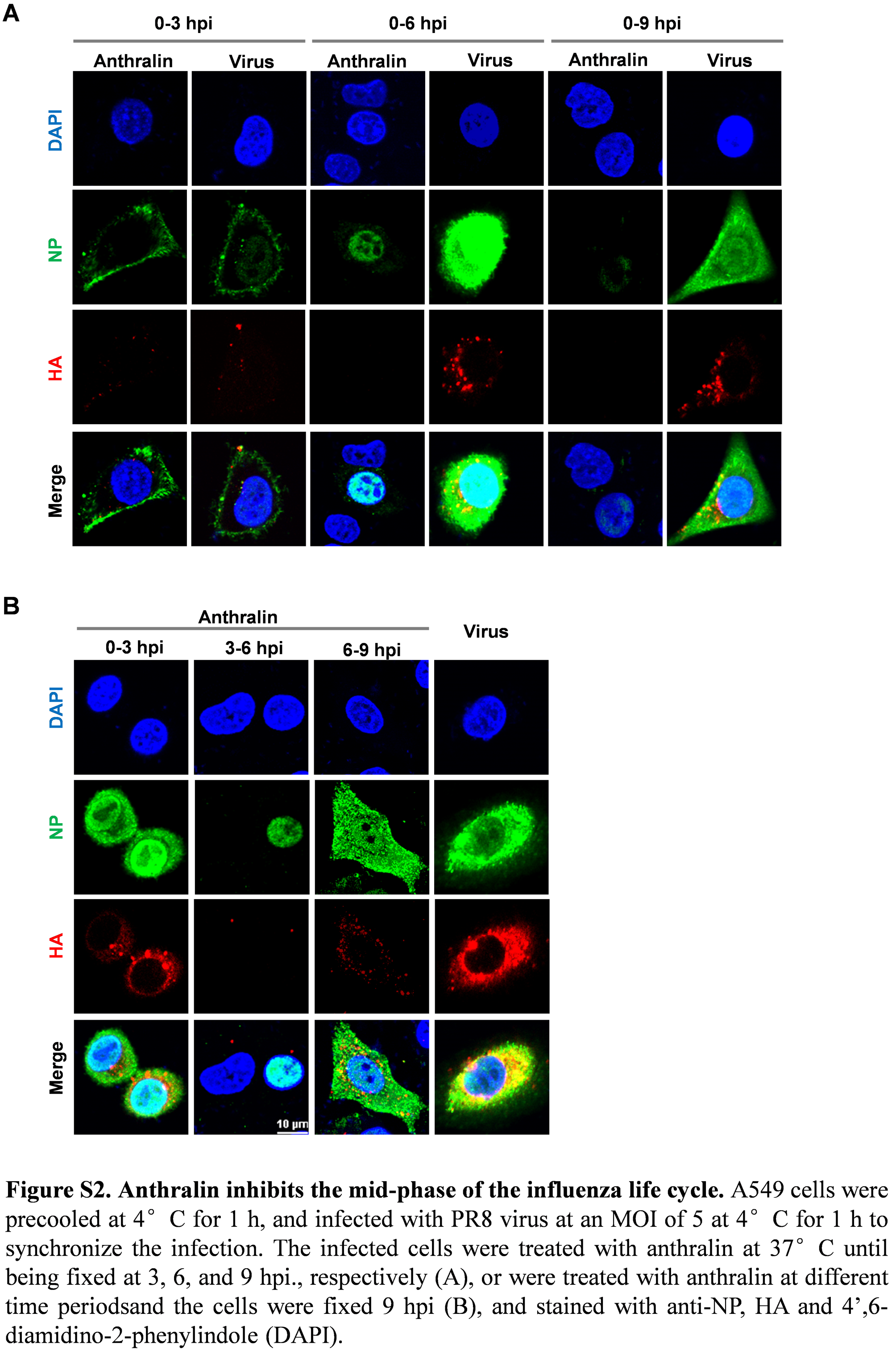

Supplement: Supplementary file 2 [file Image_2.TIF]
